# Factors influencing the use of supervised delivery services in Garu-Tempane District, Ghana

**DOI:** 10.1186/s12884-019-2295-6

**Published:** 2019-04-27

**Authors:** John Kuumuori Ganle, Mathew Loyarl Kombet, Leonard Baatiema

**Affiliations:** 1 Department of Population, Family and Reproductive Health, School of Public Health, University of Ghana, P.O.Box LG 13, Legon, Accra, Ghana; 2 Stellenbosch Institute for Advanced Study, Stellenbosch University, Stellenbosch, South Africa; 3 Regional Institute for Population Studies, University of Ghana, Legon, Accra, Ghana

**Keywords:** Maternal health, Supervised delivery, Postpartum women, Retrospective study, Ghana

## Abstract

**Background:**

There is evidence that supervised delivery has the potential to improve birth outcomes for both women and newborns. However, not all women especially in low-income settings like Ghana use supervised delivery services during childbirth. The purpose of this study was to estimate the prevalence of supervised delivery and determine factors that influence use of supervised delivery services in a local district of Ghana.

**Methods:**

A retrospective cross-sectional survey of 322 randomly sampled postpartum women who delivered between January and December 2016 in the Garu-Tempane District was conducted. Structured questionnaires were used to collect data. Descriptive, binary and multivariate logistic regression analysis techniques were used to analyse data**.**

**Results:**

Although antenatal care attendance among respondents was very high 291(90.4%), prevalence of supervised birth was only 219(68%). More than a quarter 103(32%) of the postpartum women delivered their babies at home without skilled birth attendants. After controlling for possible confounders in multivariable logistic regression analyses, factors that strongly independently predicted supervised delivery were religion (*p* < 0.01), distance to health facility (*p* < 0.05), making at least 4 antenatal care visits (*p* < 0.01), national health insurance scheme registration (*p* < 0.01), satisfaction with services received during antenatal care (*p* < 0.01), need partner’s approval before delivering in health facility (p < 0.01), woman’s thoughts that her religious beliefs prohibited health facility delivery(*p* < 0.01), and woman’s belief that there are norms in her community that did not support health facility delivery (p < 0.01).

**Conclusion:**

There is need for targeted interventions, including community mobilization and health education, and male partner involvement to help generate local demand for, and uptake of, supervised delivery services. Improvement in the quality of services in health facilities, including ensuring respect and dignity for service users, would also be essential.

**Electronic supplementary material:**

The online version of this article (10.1186/s12884-019-2295-6) contains supplementary material, which is available to authorized users.

## Background

Maternal mortality is one of the critical global health issues confronting many low-income countries in the world today. Globally, about 139 million births occur every year, and in 2013 alone, approximately 289,000 maternal deaths occurred [1, 2]. Maternal mortality remains high especially in the majority of sub-Saharan African countries, including Ghana. In 2015, sub-Saharan Africa’s maternal mortality ratio (MMR) was estimated to be 542 per 100,000 live births compared to high-income regions where MMR was 12 per 100,000 live births [1].

Utilization of supervised delivery services is one of the key proven interventions that could reduce maternal death, because of its potential to ensure safe birth, reduce both actual and potential complications and increase the survival of mothers and newborns [3]. However, a significant proportion of deliveries in low-income countries still take place at home without skilled birth attendants [1, 3]. For instance, a study done in Ethiopia revealed that as high as 71% of women received antenatal care (ANC) from a health professional; yet only 16% of deliveries were assisted by health professionals [4].

In Ghana, numerous maternal health interventions implemented in recent years such as free delivery services, establishment of community-based health planning services (CHPS), national health insurance scheme (NHIS), and health education on benefits of utilization of maternity services were expected to expand access to skilled delivery services to all pregnant women in the country [5–7]. However, this is not the case in many settings. While uptake of ANC has remained high, utilization of skilled delivery services is still relatively low. Results from Ghana’s most recent Demographic and Health Survey (GDHS) indicate that national ANC coverage of at least four visits was 97% compared to supervised delivery of 74% [8]. The situation is even worse in rural Ghana where supervised delivery is 59% compared to an urban rate of 90% [8, 9].

Women’s decisions about the choice of place of birth are influenced by many factors, from demographic, socio-economic circumstances, cultural to health system factors [2, 10–18]. In the context of Ghana more specifically, some studies have examined the factors influencing utilization of skilled delivery services as well as barriers to uptake of skilled maternal healthcare services [11, 19–25]. However, many of these studies are qualitative, and therefore do not usually provide quantitative estimates of skilled delivery prevalence and the significant determinants of skilled delivery services at the local district level. The GDHS does provide quantitative estimates of skilled delivery rates in different regions of the country. However, apart from the fact that the GDHS often mask important differences especially between districts and sub-districts, the information provided in the GDHS is often difficult to contextualise. This is particularly so because of marked differences in socio-economic and cultural circumstances across many societies in Ghana. This means that more community-based empirical research is needed to better illuminate what pertains in local districts. At the time of this study, we were not aware of any previous studies pertaining to factors that influence use of supervised delivery among women of reproductive age in the Garu-Tempane district. The purpose of this study was to estimate the prevalence of supervised delivery and determine factors that influence use of supervised delivery services among women who gave birth between January and December 2016 in the Garu-Tempane District.

## Methods

This was a retrospective cross-sectional quantitative survey, involving 322 randomly sampled postpartum women who delivered between January and December 2016. Structured questionnaires were used to collect data.

The study was conducted in the Garu-Tempane district, one of the districts in the Upper East Region of Ghana. The district is located in the south-eastern part of the region, covering an area of 1060.91 square/km and shares boundaries with Bawku Municipality to the north; Bunkpurugu- Yunyoo District to the south; Bawku West District to the west; and the Republic of Togo to the east. In 2017, the population of the district was estimated to be 218,723, constituting 1.2% of the population in the Upper East Region [9]. Over 95% of the population is rural [26]. The district has a sex ratio of 91.2 males to 100 females; a total fertility rate of 3.9; a crude birth rate of 22.7 per 1000 population; and a crude death rate of 9.89 per 1000 population [26]. The district also has a general fertility rate of 106.2 births per 1000 among women aged 15–49 years, and this is the second highest in the Upper East region [26].

The majority of the people in the district are farmers (85.2%). The dominant religions are Islam (41%), Christianity (39.7%) and Traditional African religion (16%) [26].

In relation to health, the district has a total of 47 health facilities, including one (1) private hospital which offers 24 h comprehensive emergency obstetric and newborn care services, and ten (10) public health centres, which provide basic maternal and child health services including uncomplicated birth services [9].

### Study population

The study population was postpartum women who delivered between January 2016 and December 2016 regardless of whether delivery was attended by skilled or unskilled attendants, and who were attending child welfare clinics.

### Sample size and sampling procedures

Between 2013 and 2016, an average of 72.1% of women were estimated to have given birth in health facilities with skilled attendants in the Garu-Tempane district [9]. To estimate a minimum sample size that will allow for any significant statistical association between the outcome variable and independent variables to be detected, we assumed that 72% of the women who gave birth between January and December 2016 in the Garu-Tempane District had supervised birth. Based on this assumed prevalence of supervised birth, and assuming a confidence level of 95%, a statistical power of 80%, and a 5% margin of error, we estimated a sample size of 327 using Cochran’s statistical formula [27].

In terms of sampling, a multistage sampling procedure was followed. First, the study participants were recruited from one sub-district (Kpikpira), which was randomly selected from a total of nine sub-districts. This subdistrict, however, shared similar characteristics with the eight other subdistricts. Second, all the child welfare clinics in the Kpikpira sub-district were visited. A simple random sampling procedure was then used to select individual postpartum mothers with children born between January and December, 2016, who attended the child welfare clinics. All the child welfare clinics have registers that contain the names of mothers who attend the clinic for child welfare services. The register for each clinic was obtained and all mothers with eligible children were listed and given numbers (e.g. W1, W2 …Wn). Based on this information, a google-based random number generator was used to randomly select the required number of respondents (i.e. 327). Third, the researchers visited the clinics to first meet each of the randomly selected mothers individually on the days that such women were attending. During this first meeting, the purpose of the study as well as the sampling procedures were thoroughly explained to each woman. The women were then allowed up to one week to discuss their participation in the study with their partners and other family members and to decide whether to participate or not. All selected women were re-contacted via telephone after the one-week period. Where a selected mother was not willing to participate in the study (and there were only two cases), the selection procedure was repeated to get a replacement. Finally, all women who agreed to participate in the study were individually interviewed. Interviews took place at different places including at the clinic, the woman’s home and in the market. Each woman decided on the place of interview.

### Data collection

Structured questionnaires were used to collect data (see additional file 1). The questionnaire was prepared using validated questions from the 2014 GDHS [8]. We however went beyond the GDHS questions to include a number of contextually relevant questions from one previous study in Kenya [16]. These questions were not captured in the 2014 GDHS. Examples included questions related to spousal involvement in choosing the place of birth, need to seek partner’s approval before giving birth in a health facility, whether mother-in-law decided the place of birth, distance to health facility, satisfaction with ANC services received during pregnancy, whether respondent received advice during ANC on where to give birth, and presence of norms in community prohibiting health facility birth.

The questionnaire was pre-tested in Woriyanga sub-district, one of the eight remaining sub-districts not included in this study but which had population characteristics very similar to Kpikpira sub-district. Relevant revisions were made to the instrument before use in the final data collection. The data were collected within a period of six weeks (i.e. February–March, 2017). The data collection instrument was administered personally by one of the researchers (MLK).

### Data entry and processing

The administered questionnaires were first manually examined for completeness, then hand-coded and entered into Microsoft Excel. To ensure data quality, the data was double entered into excel by the second author (MLK) and another independent person. The first author (JKG) then compared the two data entries. Errors and inconsistencies that were detected were discussed and resolved before a single database was created and exported into Stata 14 version software for further cleaning. Cleaning of the data was done by running frequencies on each variable. This checked inconsistently coded data. All inconsistently coded data were double checked with raw data from the questionnaire, and all inconsistencies and errors were resolved.

### Variables

The main outcome variable of interest in this study was supervised delivery, which was measured by the presence of a skilled birth attendant during birth and coded for the final analysis as a dichotomous categorical variable. Generally, we followed the WHO’s definition of skilled birth attendant: “an accredited health professional such as a midwife, doctor or nurse who has been educated and trained to proficiency in the skills needed to manage uncomplicated pregnancies, childbirth and the immediate postnatal period, and in the identification, management and referral of complications in women and newborns” [2]. In Ghana, all pregnant women are expected to go to a health facility for skilled birth attendants (SBAs) during birth. Therefore, we defined and measured supervised delivery as birth that occurred in any accredited public or private health facility under the supervision of a nurse, midwife or doctor. We were, however, unable to determine whether the nurses, midwives and doctors who attended the births of the women in our study were trained to proficiency in the skills needed to manage uncomplicated pregnancies, childbirth and the immediate postnatal period, and in the identification, management and referral of complications in women and newborn. While staff shortages in rural areas can sometimes compel other health staff like general nurses or medical practitioners who may not have been originally trained in midwifery to attend to women in labour, we assumed that the health staff who attended to women in our study had some amount of training sufficient to manage normal births and to refer complicated ones. Birth outside a health facility under the supervision of a traditional birth attendant (TBA) or family member was considered unsupervised birth.

Several independent variables were also measured, including socio-demographic factors such as age, maternal education, occupation, marital status, religion, husband’s occupation, and place of residence; health facility level factors such as distance to health facility, satisfaction with service, NHIS registration, and advice on where to deliver during ANC; socio-cultural factors such as women’s decision-making on place of birth, and presence of cultural norms prohibiting or supporting skilled delivery. One independent variable that presented measurement difficulties was distance. We collected data on geographical distance by road as measured by how far (in kilometers) a respondent lived away from the health facility where supervised delivery took place or would have taken place in the case of women who gave birth at home. This ranged from under half a kilometer to over 20 km. We also collected data on different modes of transportation, and the time it took to reach the facility. Modes of transportation ranged from walking, being picked on a bicycle, a donkey, a motor-bike, a motor-tricycle, to use of public transport (minibus services). As a result, travel time varied between under 20 min to several hours. We also collected data on cost of travel, albeit many respondents who walked or used personal bicycles and donkeys could not quantify their cost of travel in actual monetary terms. From all the measures related to travel, geographical distance was the most reliable measure compared to mode of travel, travel time or cost. We therefore used geographic distance to the facility where birth took place or would have taken place. For ease of analysis, we categorized respondents into two: those who traveled or would have traveled between 0 and 10 km and those who traveled 11+ (this ranged from 11 to 25 km).

### Statistical analysis

Descriptive statistical analysis such as frequency and percentage distribution were first used to describe characteristics of participants and prevalence of supervised delivery. Inferential statistical analyses such as bivariate, binary and multivariate logistic regression analyses were used to examine factors associated with utilization of skilled delivery. Odd ratios were also estimated. A confidence of 95% was used, and a *p* < 0.05 was considered statistically significant.

## Results

### Socio-demographic characteristics

Table 1 presents information on the socio-demographic characteristics of respondents. Out of the 327 questionnaires that were administered, 322 were used for analysis. We excluded five questionnaires because the deliveries occurred en-route to the health facility. The majority of respondents (67, 20.8%) were from age group 25–29 years. Majority of respondents (165, 51.2%) had no formal education. The majority of the respondents (103, 32%) reported farming as their main source of livelihood. Furthermore, majority of the respondents (271, 84.2%) resided in rural areas, while 291(90.4%) of the respondents indicated that they made at least 4 ANC visits during their most recent pregnancy.

**Table 1:** Socio-demographic characteristics of respondents (*n* = 322)

Characteristics	Frequency	Percent (%)
Age		
15–19	33	10.2
20–24	65	20.2
25–29	67	20.8
30–34	51	15.8
35–39	46	14.29
40–44	30	9.3
45–49	30	9.3
Level of education		
No formal education	165	51.2
Primary	70	21.7
JHS/Middle School	46	14.3
Secondary	24	7.5
Tertiary	17	5.3
Marital status		
Married	289	89.8
Divorced	12	3.7
Separated	6	1.9
Co-habiting	1	0.3
Single	14	4.3
Type of Marriage		
Monogamous	170	58.8
Polygamous	119	41.2
Religion		
Christianity	146	45.3
Islamic	141	43.8
Traditional	35	10.9
Occupation		
Farming	103	32.0
Trading	40	12.4
Housewife	80	24.8
Hairdresser	26	8.1
Civil/Public servant	18	5.6
Student	9	2.8
Other	46	14.3
Residence		
Urban	13	4.0
Peri-Urban	38	11.8
Rural	271	84.2
Made at least 4 ANC visits		
Yes	291	90.4
No	31	9.6

### Prevalence of supervised delivery

**Fig. 1:**
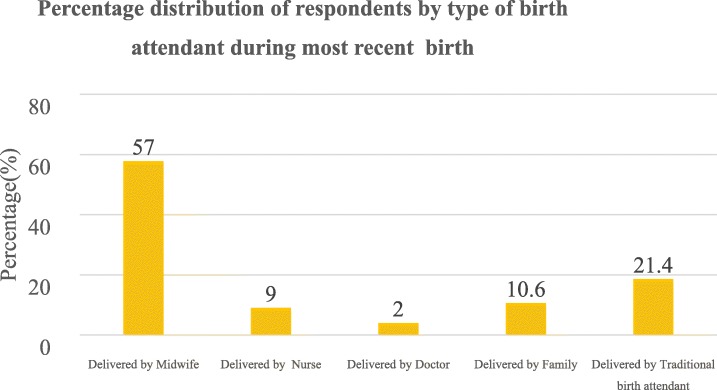
Type of birth attendant during most recent birth

The majority of the respondents (99, 30.4%) delivered their most recent baby within 4–6 months prior to the study. More than a quarter (103, 32%) of the women had unsupervised delivery. Figure 1 further shows disaggregated data on the type of birth attendant. Among the 68% of women who had supervised delivery, majority (125, 57%) was attended to by midwives. Only 2% of them were attended to by doctors. Finally, among the 103(32%) who had unsupervised birth, majority (22, 21.4%) were delivered by TBAs.

### Factors influencing supervised delivery

Table 2 shows the results of bivariate analysis investigating association between 18 different variables and the outcome of interest (supervised delivery). Socio-demographic factors such as age (*p* < 0.01), religion (p < 0.01), partner’s occupation (p < 0.01) were all statistically associated with use of supervised delivery. However, education level of respondents (*p* = 0.287), marital status (=0.087), type of marriage (*p* = 0.072), respondents’ occupation (*p* = 0.067) and place of residence (*p* = 0.063) were not significantly associated with skilled delivery.

**Table 2:** Factors associated with use of supervised delivery (bivariate)

Variable	Had supervised delivery		Chi-square *P*-value
	No N(%)	Yes N(%)	
Age			
15–19	13(39.4%)	20(60.6%)	0.000*
20–24	12(18.5%)	53(81.5%)	
25–29	15(22.4%)	52(77.6%)	
30–34	5(9.8%)	46(90.2%)	
35–39	14(30.0%)	32(69.5%)	
40–44	21(70%)	9(30.0%)	
45–49	22(73%)	8(26.6%)	
Educational level			
No formal education	55(33.3%)	110(66.7%)	0.287
Primary school	16(22.9%)	54(77.1%)	
JHS/Middle school	15(32.6%)	31(67.4%)	
Secondary school	11(46.0%)	13(54.0%)	
Tertiary	5(29%)	12(71.0%)	
Marital status			
Married	83(29%)	206(71%)	0.087
Divorced	7(58%)	5(42%)	
Separated	2(33%)	4(67%)	
Co-habiting	1(100%)	–	
Single	9(64%)	5(36%)	
Type of marriage			
Monogamous	41(24%)	129(76%)	0.072
Polygamous	43(36%)	76(64%)	
Religion			
Christianity	20(14%)	90(86%)	0.000*
Islamic	65(46%)	76(54%)	
Traditional	17(49%)	18(51%)	
Occupation			
Farming	13(13%)	90(87%)	0.067
Hair dresser	8(31%)	18(69%)	
Housewife	40(50%)	40(50%)	
Seamstress	16(35%)	26(65%)	
Trading	10(25%)	30(75%)	
Civil/Public servant	5(28%)	13(72%)	
Student	7(78%)	22(22%)	
Others	3(75%)	1(25%)	
Partner’s occupation			
Trading	27(40%)	40(60%)	0.001*
Civil servant	6(19%)	26(81%)	
Farming	45(27%)	123(73%)	
Student	1(6%)	15(94%)	
Others	5(83%)	1(17%)	
Residence			
Urban	1(8.0%)	12(82.0)	0.063
Peri-urban	8(21.0%)	30(79.0%)	
Rural	93(34.0%)	178(66.0%)	
Made at least 4 ANC visits			
Yes	80(27.49)	211(72.51)	0.000*
No	22(70.97)	9 (29.03)	
Received advice on where to deliver at the ANC			
Yes	67(26.38%)	187(73.62%)	0.439
No	12(32.43)	25(67.57)	
Satisfaction with ANC service received during pregnancy			
Very satisfied	9(19.15%)	38(80.85%)	0.000*
Satisfied	20(15.63%)	108(84.38%)	
Dissatisfied	13 (44.83%)	16 (55.17%)	
Very dissatisfied	14(87.5%)	2(12.5%)	
Distance to health facility			
0-10 km	42(41.18)	130(59.09)	0.003*
11 + km	60(58.82)	90(40.91)	
NHIS Registered			
Yes	50(22%)	174(78%)	0.000*
No	52(53%)	46(47%)	
Discussed with partner where to deliver			
Yes	65(59.6%)	185(86.8%)	0.000*
No	44(40.4%)	28(13.1%)	
Needed partner’s approval before delivering at health facility			
Yes	71(71.0%)	197(92.06%)	0.000*
No	29(29.0)%	17(7.94%)	
Thinks her religious beliefs prohibit health facility delivery			
Yes	50 (49.02%)	39 (17.73%)	0.000*
No	52 (50.98%)	181(82.72%)	
Mother-in-law decided place of delivery			
Yes	7(6.86%)	29(13.18%)	0.094
No	95(93.14%)	191(86.82%)	
Presence of norms in community prohibiting health facility delivery			
Yes	54(52.94%)	48(21.82%)	0.000*
No	48(47.06%)	172(78.18%)	

*P < 0.05

A number of health system-related factors were also found to be statistically associated with supervised delivery. These included making at least 4 ANC visits during the most recent pregnancy (*p* < 0.01), satisfaction with ANC services received during pregnancy (*p* < 0.01), distance to health facility (p < 0.01), and NHIS registration (*p* < 0.01). However, receiving advice on where to deliver during ANC (*p* = 0.439) was not statistically significantly related to supervised delivery.

In addition, socio-cultural and community level factors such as discussion with partner about place of delivery (*p* < 0.01), need partner’s approval before delivering in a health facility (p < 0.01) as well as a woman’s thoughts that her religious beliefs prohibited health facility birth (p < 0.01) or belief that there were norms in her community that did not support health facility birth (p < 0.01) were statistically significantly associated with supervised birth. Mother-in-law deciding the place of delivery was, however, not statistically significantly associated with supervised birth (*p* = 0.094).

To further investigate strength of associations, simple logistic regression showed seven factors to be strongly independently associated with supervised delivery (Table 3). Specifically, when compared to Christians, Muslims and women who practiced traditional African religion were, respectively, 5.4 times (AOR = 5.43; 95%CI = 2.39–12.35) and 9.5 times (AOR = 9.51; 95%CI = 2.90–31.20) more likely not to utilize supervised delivery during their most recent birth. When compared to women who lived within 0-10 km from a health facility, those who lived 11+ away from a health facility were almost twice more likely not to use supervised delivery services (AOR = 1.97; 95%CI = 1.14–3.42). Women who made less than 4 ANC visits were almost 5 times more likely not to use supervised delivery services when compared with those who made at least 4 ANC visits (AOR = 4.86; 95%CI = 1.78–13.2). Similarly, women who were not NHIS registered, when compared to those who were registered, were 4.42 times more likely not to use supervised delivery services (AOR = 4.418; 95%CI = 2.529–7.718). Finally, women who said they needed their partner’s approval before delivering in a health facility were about 4.5 times more likely not to use supervised delivery services when compared with those who said they did not require such permission (AOR = 4.523; 95%CI = 2.106–9.714).

**Table 3:** Factors associated with use of supervised delivery (Logistic)

Variable	Crude		Adjusted	
	OR	95% CI	OR	95% CI
Age^a^				
15–19	*Ref*			
20–24	0.35	0.12–0.89	0.57	0.191–1.70
25–29	0.44	0.21–0.18	0.61	0.21–1.80
30–34	0.17	0.05–0.53	0.28	0.08–1.02
35–39	0.67	0.26–1.72	0.81	0.26–2.54
40–44	3.59	1.26–10.23	5.11	1.51–17.3
45–49	4.23	1.50–12.32	6.81	1.9–23.5
Religion^b^				
Christianity	*Ref*			
Islamic	5.39	3.03–9.59	5.43	2.39–12.35
Traditional	15.88	5.95–2.64	9.51	2.90–31.20
Partner’s Occupation^c^				
Farming	*Ref*			
Trading	1.845	1.016–3.347	1.436	0.741–2.784
Civil/Public servant	0.630	0.243–1.632	0.390	0.112–1.354
Student	0.182	0.023–1.419	0.160	0.019–1.311
Others	13.666	1.554–20.17	10.585	1.054–16.24
Distance to health facility^d^				
0-10Km	*Ref*			
11 + Km	2.06	1.280–3.325	1.971	1.138–3.415
Made at least 4 ANC visits^e^				
Yes	*Ref*			
No	6.45	2.85–14.59	4.86	1.78–13.2
Thinks her religious beliefs prohibit health facility delivery^f^				
Yes	*Ref*			
No	0.224	0.133–0.376	0.258	0.59–0.92
Satisfaction with ANC service received^g^				
Very satisfied	*Ref*			
Satisfied	0.78	0.33–1.87	0.65	0.23–1.86
Dissatisfied	3.43	1.22–9.621	4.56	1.33–15.61
Very dissatisfied	29.56	5.68–153.92	31.84	3.09–38.36
NHIS Registered^h^				
Yes	Ref			
No	3.933	2.371–6.526	4.418	2.529–7.718
Discussed with your partner where to deliver^i^				
Yes	*Ref*			
No	*5.73*	2.96–11.09	2.11	0.64–6.93
Needed partner’s approval before delivering at health facility^j^				
Yes	Ref			
No	4.73	2.452–9.133	4.523	2.106–9.714
Presence of norms in community prohibiting health facility delivery^k^				
Yes	*Ref*			
No	0.25	0.14–0.41	0.259	0.10–0.67

^a^adjusted for religion, partner’s occupation, made at least 4 ANC visits, distance to health facility, NHIS registration, discussed with partner where to deliver, needed partner’s approval before delivering at health facility, satisfaction with services received during ANC, thinks her religious beliefs prohibit health facility delivery, and presence of taboos in community prohibiting health facility delivery; ^**b**^adjusted for age, partner’s occupation, made at least 4 ANC visits, distance to health facility, NHIS registration, discussed with partner where to deliver, needed partner’s approval before delivering at health facility, satisfaction with services received during ANC, thinks her religious beliefs prohibit health facility delivery, and presence of taboos in community prohibiting health facility delivery; ^**c**^adjusted for age, religion, made at least 4 ANC visits, distance to health facility, NHIS registration, discussed with partner where to deliver, needed partner’s approval before delivering at health facility, satisfaction with services received during ANC, thinks her religious beliefs prohibit health facility delivery, and presence of taboos in community prohibiting health facility delivery; ^**d**^adjusted for age, religion, partner’s occupation, made at least 4 ANC visits, NHIS registration, discussed with partner where to deliver, needed partner’s approval before delivering at health facility, satisfaction with services received during ANC, thinks her religious beliefs prohibit health facility delivery, and presence of taboos in community prohibiting health facility delivery; ^**e**^adjusted for age, religion, partner’s occupation, distance to health facility, NHIS registration, discussed with partner where to deliver, needed partner’s approval before delivering at health facility, satisfaction with services received during ANC, thinks her religious beliefs prohibit health facility delivery, and presence of taboos in community prohibiting health facility delivery; ^**f**^adjusted for age, religion, partner’s occupation, made at least 4 ANC visits, distance to health facility, NHIS registration, discussed with partner where to deliver, needed partner’s approval before delivering at health facility, satisfaction with services received during ANC, and presence of taboos in community prohibiting health facility delivery; ^**g**^adjusted for age, religion, partner’s occupation, made at least 4 ANC visits, distance to health facility, NHIS registration, discussed with partner where to deliver, needed partner’s approval before delivering at health facility, thinks her religious beliefs prohibit health facility delivery, and presence of taboos in community prohibiting health facility delivery; ^**h**^adjusted for age, religion, partner’s occupation, made at least 4 ANC visits, distance to health facility, discussed with partner where to deliver, needed partner’s approval before delivering at health facility, satisfaction with services received during ANC, thinks her religious beliefs prohibit health facility delivery, and presence of taboos in community prohibiting health facility delivery; ^**i**^adjusted for age, religion, partner’s occupation, made at least 4 ANC visits, distance to health facility, NHIS registration, needed partner’s approval before delivering at health facility, satisfaction with services received during ANC, thinks her religious beliefs prohibit health facility delivery, and presence of taboos in community prohibiting health facility delivery; ^**j**^adjusted for age, religion, partner’s occupation, made at least 4 ANC visits, distance to health facility, NHIS registration, discussed with partner where to deliver, satisfaction with services received during ANC, thinks her religious beliefs prohibit health facility delivery, and presence of taboos in community prohibiting health facility delivery; ^**k**^adjusted for age, religion, partner’s occupation, made at least 4 ANC visits, distance to health facility, NHIS registration, discussed with partner where to deliver, needed partner’s approval before delivering at health facility, satisfaction with services received during ANC, and thinks her religious beliefs prohibit health facility delivery.

*OR* odds ratio, *CI* confidence interval, *ref* reference category

## Discussion

This study is one of the first to focus on examining the factors that influence use of supervised delivery services among postpartum women in Garu-Tempane District of Ghana. Findings suggested that while ANC coverage was generally high (90.4%), the prevalence of supervised delivery was still relatively low (68%). After accounting for possible confounders, religion, distance to health facility, making at least 4 ANC visits, NHIS registration, satisfaction with services received during ANC, need partner’s approval before delivering in a health facility, woman’s thoughts that her religious beliefs prohibited health facility delivery, and woman’s belief that there were norms in her community that did not support health facility delivery were the seven factors that strongly predicted supervised delivery.

Several findings from our study are similar to findings from previous studies. For example, women aged below 20 years (20, 60.6%) were the least users of supervised birth in a health facility. This is consistent with the 2014 GDHS, which suggests that pregnant women between the ages of 20 and 34 years were more likely to use a health facility for delivery [8]. With respect to religion, Christian women (90, 86%) were more likely to use supervised delivery services as compared with those who profess Islamic (76, 54%) and traditional (18, 51%) faith. The findings of our study are consistent with a study by Gyimah and colleagues in Ghana, which indicated high uptake of institutional delivery services by Christian women as compared to women who profess Islamic and traditional faith [28]. Similar findings have also been previously reported by Stephenson et al. [10].

Taken together, the results of this study suggest that although supervised delivery services may be free in Ghana, a number of socio-demographic, health system and socio-cultural factors still prevent women from utilizing such services. As shown in this study, although there was little statistical difference in supervised delivery rates in relation to age once other factors like religion were accounted for, it is very clear that women under 19 years and those above 39 years were the least users of supervised delivery services as compared to those between the ages of 20 and 39 years. Different factors may interplay to explain this especially in the context of Garu-Tempane. Possible explanations could include the fact that expectant mothers below 19 years are most unlikely to be in stable relationships, lack skills for employment and may be stigmatized both in the community and at the health facility for getting pregnant [7, 11]. This is more likely given that 18 years is the legal age of marriage in Ghana. The overall effect is that, they may not be able to afford the cost related to birth in health facilities such as transportation charges, opportunity cost and other unofficial expenses. Also, for women above 39 years, it may be due to perceived maternity experience and confidence acquired from previous births. Women above 39 years are also more likely to be multiparous with a large family size, giving an indication of possible financial and resource constraints on the family income, and this may be compounded when the woman does not earn income on her own.

Religious beliefs and other associated cultural beliefs and norms are known factors affecting skilled delivery. In this study, both religious affiliation and a woman’s thought that her religious beliefs prohibited health facility delivery, independently predicted non-use of supervised delivery. Previous studies, as did ours, have shown that women who reported that there were norms in their communities that did not support health facility birth were at increased odds of not having had supervised delivery during their most recent birth. This result is however not surprising as previous studies have shown that most societies in northern Ghana are deeply rooted in culture and therefore women are expected to respect even unwholesome cultural practices that govern reproductive behaviour and which may directly or indirectly affect their health [23, 25]. This result would suggest a need for continuous community education and engagement to address cultural norms that do not support supervised delivery.

Another inter-related factor to religion and cultural norms is decision-making at the family level. The role of men in facilitating or discouraging women’s access to essential reproductive and maternal healthcare services in low-income settings is increasingly being recognized [23, 25, 29–32]. A number of recent studies in Ghana have indicated that men’s disapproval is a major barrier to women’s use of skilled maternal and newborn healthcare services [30–32]. In some parts of the world, it has equally been noted that husband’s approval is an important determinant of access and use of maternal and safe motherhood services [33–38]. It has therefore been suggested that though maternal and newborn survival requires improvements in basic and comprehensive obstetric care coverage and quality, inclusion of men in maternal and safe motherhood services is required to increase the use of these services, eliminate delays in accessing care, and promote timely referral when problems arise [30–32, 39]. Clearly, our results support greater male involvement. Innovative engagement strategies beyond the provision of money, arranging transport or assisting in household chores would be particularly critical. For example, invitation cards could be used to invite men to routine ANC classes and pregnancy schools to enhance men’s understanding of maternal and safe motherhood issues as well as the importance of supervised delivery. This has been shown elsewhere to enhance male involvement and health outcomes [40].

Distance to health facilities in this study was shown to be a predictor of utilisation of supervised delivery services. Distance can be measured in several ways: it is a combination of geographical distance, mode of travel, time of travel, and costs of travel. While we focused on geographical distance in this study, we acknowledge mode of travel, time it takes to travel, and cost of travelling to reach the facility are all important. For instance, none of the respondents mentioned ambulance as their means of transport to the health facility. This is understandable because there are no functional ambulances apart from the district hospital. Thus, most respondents relied on personal means of transport, many of which (e.g. bicycle and donkey) were actually inappropriate for conveying a pregnant woman in labour. Therefore, women and their families will most likely make decisions about supervised delivery service utilization after careful consideration of factors such as mode of travel, time it takes to travel, and the cost of travelling to reach the facility, and the difficulty of arranging an appropriate means of transport. However, our focus on geographical distance to health facilities has helped to highlight the crucial fact that supervise delivery services are not readily available to women in their communities. The situation appears to be worse in the context of the Garu-Tempane district where the road network is poor and generally inaccessible by cars especially during the rainy season.

This study has also shown that other health service-related factors such being registered on the NHIS play a significant role in the use of supervised delivery services. Since 2003, Ghana has operated a user-fee exemption policy for institutional delivery [5, 7]. However, there is evidence in many places that women still pay other informal fees [5, 41]. The situation is even worse when a woman is not registered on the NHIS. Fears that supervised delivery may generate more additional costs among women who are not registered on the NHIS may therefore explain why such women are less likely to use supervised delivery services. It is even possible that such women may have had less contact with the healthcare system in relation to other more general healthcare precisely because they are not NHIS-registered. This would suggest a need to encourage registration on the NHIS, including strengthening and ensuring effective coverage of the free delivery policy, especially in rural areas.

Also, women who were ‘very dissatisfied with ANC services received’ during pregnancy were 31.84 times more likely not to use supervised delivery services when compared with those who were very satisfied (AOR = 31.84; 95%CI = 3.09–38.36; *p* = 0.004). Poor attitudes of healthcare providers in addition to infrastructural challenges and long waiting times at health facilities are factors that contribute to satisfaction. It is a known fact that most public sector healthcare providers work under undesirable conditions especially in rural Ghana [6]. These include attending to a large number of clients and in most cases, one nurse working both day and night. These conditions may influence negative attitudes of nurses in the context of Garu-Tempane and Ghana at large, often exhibited by health providers in the form of anger, negative expressions and poor provider-client relationships. This is, however, not a typically Ghanaian issue, but a general issue in overburdened health systems with a human resource crisis [10]. Unsatisfactory services received during pregnancy may however be a deterrent to the use of supervised delivery for women. This would also suggest a need for some continuous training of healthcare providers in client-centred communication and service provision, in addition to better health workforce planning to reduce workload for healthcare providers and overcrowding in ANC clinics and maternity wards.

Finally, we are surprised that some factors were not statistically associated with supervised delivery in our study. The finding that maternal education was not a statistically significant factor is contrary to many studies in Ghana [15, 21, 28]. We think that it is probably because of the relatively high representation of women with no formal education (165, 51%) in our study. Also, advice received on where to deliver at ANC was not significantly associated with supervised delivery in this study. This is also counter intuitive because one would expect counselling and advise on the need to seek supervised delivery during ANC to positively impact on women’s decision to receive supervised delivery during childbirth [17]. A possible reason could be missed opportunity at ANC to adequately counsel and educate women on the need for skilled care at birth. Also, unsatisfactory attitudes of some healthcare providers at ANC as well as other economic and social considerations might have accounted for non-usage of supervised delivery even if advice and counselling on place of delivery were provided. Lastly, the influence of mothers-in-law on place of birth was not significantly associated with supervised delivery in this study. This is in contrast with a number of previous studies in northern Ghana [11, 19, 25]. We think that it is probably because women’s maternal health seeking behaviours in the study area are greatly influenced by husbands and other male family heads rather than mothers-in-law, as illustrated by the strong positive statistical association between the need for partner’s approval and less use of supervised delivery.

The results of our study should, however, be interpreted in view of certain limitations. The retrospective nature of the design meant that respondents had to recall events that may have taken place several months back. There is therefore the possibility of recall bias on the part of respondents. Also, the use of a structured questionnaire alone to elicit responses from women impaired women’s ability to explain or give qualitative accounts for non-use of skilled delivery. This implies that a further qualitative study might be required to supplement our preliminary quantitative work. Also, our study focused on a selected population – postnatal women who were attending child welfare clinics. Therefore, women who do not visit these child welfare clinics may have been missed in our study. In addition, the study covered only one sub-district. Therefore, the limitation of generalising the results is acknowledged. These limitations notwithstanding, important lessons could be learned to inform policy and practice.

## Conclusions

This study has helped to reveal important factors influencing supervised delivery services among postpartum women in the Garu-Temapne district. The results particularly suggest a need to focus on addressing factors that discourage uptake of skilled delivery services. In particular, the results call for more interventions to increase the prevalence of skilled delivery in the district and the country at large, which is the most effective way of reducing maternal morbidity and mortality. In this regard, it is important that the Ghana health service ensures essential maternal health interventions target women such as those aged below 20 and above 40, among whom prevalence of supervised delivery is low. This could help to promote equitable use of supervised delivery services and equitable maternal and newborn health outcomes. In addition, improving the quality of care in health facilities is critical. This could be done by increasing resource allocation to boost supply of essential consumables, expanding the human resource base to reduce overwork, and routine on-the-job training of service providers especially in interpersonal communication skills and patient-centred care, including ensuring respect and dignity for women who make use of skilled delivery services in health facilities. Finally, community mobilization and engagement, especially male involvement, should be considered in addition to other health delivery strengthening interventions like cultural competence and client-centred training for maternal healthcare providers.

## Additional file

Additional file 1: Study Questionnaire. (DOCX 85 kb)

## Abbreviations

ANC: Antenatal Care
CHPS: Community-based Health Planning Services
GDHS: Ghana Demographic and Health Survey
MMR: Maternal Mortality Ratio
NHIS: National Health Insurance Scheme
SBA: Skilled Birth Attendant
TBA: Traditional birth attendant
WHO: World Health Organization
