# Supplementary material for: Factors influencing the use of supervised delivery services in Garu-Tempane District, Ghana
Source: BMC Pregnancy Childbirth. 2019 Apr 27;19:141. doi: 10.1186/s12884-019-2295-6 (PMC6487062; doi:10.1186/s12884-019-2295-6)
Supplement: Supplementary file 1 — Study Questionnaire. (DOCX 85 kb) [file 12884_2019_2295_MOESM1_ESM.docx]

**SCHOOL OF PUBLIC HEALTH**

**UNIVERSITY OF GHANA- LEGON**

**STUDY QUESTIONNAIRE**

My name is ……………………………….. I am working for the School of Public Health, University of Ghana. We are conducting a research to understand the **Factors influencing the use of supervised delivery services in Garu-Tempane District**. The information we collect will help understand how to plan for skilled delivery services for pregnant women and their families in this district. You have been randomly chosen to participate in the study; and we would like to ask you some questions. The questions usually take about 20-40 minutes. All the answers you give will be confidential and will not be shared with anyone other than members of the study team. You do not have to participate in the survey; but we hope you will agree to answer the questions since your views are important. If I ask you any question you do not want to answer, just let me know and I will go on to the next question or you can stop the interview any time.

In case you need more information about the survey, you may contact the persons listed on the information sheet that has already been given to you.

Do you have any questions?

| May I have your permission to ask you questions, and are you willing to participate? | Yes [ ].  No [ ]  Not Now [ ] | 1  2  3 |
| --- | --- | --- |
| If no, may I ask why you’re not willing to participate?  I**NTERVIEWER**: DO NOT ASK ANY FURTHER QUESTIONS TO RESPONDENT AND THANK HER FOR HER TIME | I don’t have any time [ ]  My spouse/partner disapproves my participation [ ]  I do not want to talk about my health and life experiences [ ]  I do not want to talk to you [ ]  Other; please specify………………………………… | \| 1 \| \| --- \| \| 2 \| \| 3 \| \| 4 \| \| 5 \| \|  \| \|  \| |
| If it is not a good time to talk, when would be more suitable for you?  I**NTERVIEWER**: DO NOT ASK ANY FURTHER QUESTIONS TO RESPONDENT AND THANK HER FOR HER TIME | Date:……………………………………..  Time:…………………………………….  Venue:…………………………………… | |

Interview Date: …………………………………

ID No …………………………………………

**A. SOCIO-DEMOGRAPHIC CHARACTERISTICS**

|  | QUESTION | RESPONSE | CODE |
| --- | --- | --- | --- |
| Q1 | How old are you? |  |  |
| Q2 | What is your highest level of education? | 1. No formal education [ ]  2. Primary [ ]  3. JHS/Middle school [ ]  4. Secondary [ ]  5. Tertiary [ ] | 1  2  3  4  5 |
| Q3 | What is your marital status? | 1. Married [ ]  2. Not married [ ]  3. Divorced [ ]  4. Separated [ ]  5. Co-habiting [ ]  6. Single [ ] | \| 1 \| \| --- \| \| 2 \| \| 3 \| \| 4 \| \| 5 \|   6 |
| Q4 | If you are married, what type of marriage is it? | 1. Monogamous 2. Polygamous 3. Others (specify)….. | \| 1 \|  \|  \|  \| \| --- \| --- \| --- \| --- \| \| 2 \|  \|  \|  \| \| 3 \|  \|  \|  \| \|  \|  \|  \|  \| |
| Q5 | What is your religion? | \|  \| 1.Christianity[ ]  2.Islamic [ ]  3.Traditional [ ] \| \| --- \| --- \| | \| 1 \|  \| \| --- \| --- \| \| 2 \|  \| \| 3 \|  \| |
| Q6 | What is your occupation? | 1. Farming [ ]  2. Trading [ ]  3. House wife [ ]  4. Seamstress [ ]  5. Hair dresser [ ]  6. Public servant [ ]  7. Others (Specify)…… | \| 1 \| \| --- \| \| 2 \| \| 3 \| \| 4 \| \| 5 \|   6  7 |
| Q7 | What is your husband’s occupation? | 1. None [ ] 2. Trading [ ] 3. Civil servant [ ] 4. Student [ ] 5. Farmer [ ] 6. Others (specify)............. | \| 1 \| \| --- \| \| 2 \| \| 3 \| \| 4 \| \| 5 \|   6 |
| Q8 | Your place of residence | 1. Urban [ ]  2. Peri-urban [ ]  3. Rural [ ] | \| 1 \| \| --- \| \| 2 \| \| 3 \| |
| Q9 | Your ethnicity | 1. Kusaasi [ ]  2. Bimoba [ ]  3. Busangas [ ]  4. Moshi [ ]  5. Others (specify) ………………………. | \| 1 \| \| --- \| \| 2 \| \| 3 \| \| 4 \| \| 5 \| |

**B. COVERAGE OF SKILLED BIRTH ATTENDANCE.**

|  | QUESTION | RESPONSE | CODE |
| --- | --- | --- | --- |
| Q10 | \| When did you deliver your most recent baby? \| \| --- \| | 1. 3 months and below [ ] 2. 4 to 6 months [ ] 3. 7 to 9 months [ ] 4. 10 to 12 months [ ] | \| 1 \| \| --- \| \| 2 \| \| 3 \|   4 |
| Q11 | Where did you deliver your most recent baby? | 1. At home [ ]  2. At health facility [ ] | 1  2 |
| Q12 | Who delivered you in your most recent birth? | 1. Doctor [ ]  2. Midwife/Auxiliary midwife [ ]  3. Nurse) [ ]  4. Traditional birth attendant [ ]  5. Family member [ ] | 1  2  3  4  5 |
| Q13 | What mode was your most recent delivery? | 1. Spontaneous vaginal delivery [ ]  2. Caesarean section [ ] | 1  2 |

C. **HEALTH INSTITUTIONAL FACTORS THAT ARE RELATED TO USE OF SUPERVISED DELIVERY**

|  | QUESTIONS | RESPONSE | CODE |
| --- | --- | --- | --- |
| Q14 | Did you attend any antenatal care (ANC) during your last pregnancy? | 1.Yes [ ] if yes skip Q20  2. No [ ] if no skip to Q20 | 1  2 |
| Q15 | Why did you go for antenatal care (ANC)? Tick those applicable. | 1.To get registration card []  2. To get injection [ ]  3. To receive free medicine [ ]  4. To know if the baby/fetus is healthy or alive [ ]  5. Because it is important [ ]  6. Other reasons.………………  ……………………… | 1  2  3  4  5 |
| Q16 | Were you advised on where to deliver at the ANC? | 1. Yes [ ]  2. No [ ] | 1  2 |
| Q17 | In your own estimation, how long did you have to wait to be attended at the health facility? |  |  |
| Q18 | Which of the following was the attitude of the nurses towards you in the labor ward when you went to deliver your baby at the health facility? | 1. They used abusive language []  2. They gave me necessary attention I needed [ ]  3. They were friendly [ ]  4. Others (specific)…….. | 1  2  3  4 |
| Q19 | To what extent were you satisfied with the services you received from your delivery attendant? | 1. Very satisfied [ ] 2. Satisfied [ ] 3. Dissatisfied [ ] 4. very dissatisfied [ ] | \| 1 \| \| --- \| \| 2 \| \| 3 \| \| 4 \| |
| Q20 | Why did you not attend ANC? | 1. Lack of money [ ]  2. Not necessary [ ]  3. Inconvenient service hours [ ]  4. Access problem [ ]  5. Others (specify)…………….. | \| 1 \| \| --- \| \| 2 \| \| 3 \| \| 4 \| \| 5 \| \|  \| |
| Q21 | Why did you not deliver in health facility | 1.Better service at home [ ]  2.Access problem [ ]  3.Not necessary [ ]  4.Lack money [ ]  5.Inconvenient service hours [ ]  6. Others (specify)…………… | \| 1 \| \| --- \| \| 2 \| \| 3 \| \| 4 \| \| 5 \|   6 |
| Q22 | How far is your residence from a health facility? | 1. 0- 5km [ ]  2. 6+km [ ] | 1  2 |
| Q23 | Before delivery, did you arrange for transport to health facility? | 1.Yes [ ]  2. No [ ] if no skip to Q25 | 1  2 |
| Q24 | What kind of transport? | 1. Motor bike [ ]  2. Motor tricycle [ ]  3. Ambulance [ ]  4. Commercial vehicle [ ]  5. Others (specify)………………… | \| 1 \| \| --- \| \| 2 \| \| 3 \| \| 4 \| \| 5 \| |
| Q25 | Why did you not arrange for transport? |  |  |
| Q26 | Did you incur any additional cost despite the use of health insurance in your last delivery at the health facility? | 1.Yes [ ]  2. No [ ] | 1  2 |
| Q27 | How much were you charged for delivery? | (.......)GH |  |

**D. SOCIO-CULTURAL RELATED FACTORS**

|  | QUESTION | RESPONSE | CODE |
| --- | --- | --- | --- |
| Q28 | Who decided for you the choice of place for your delivery? | 1. Myself [ ]  2. Husband [ ]  3. Mother [ ]  4. Mother in-law[ ]  5. Siblings’ [ ]  6. friends [ ] | 1  2  3  4  5  6 |
| Q29 | Did you discuss with your partner where to deliver? | 1. Yes [ ]  2. No [ ] | 1  2 |
| Q30 | Did you have to seek your husband’s approval before deciding to deliver at the health facility? | 1. Yes [ ]  2. No [ ] | 1  2 |
| Q31 | If you decide to seek skilled attendance without your husband’s concern, what would be the consequence? | 1. My husband will punish me [ ]  2. Community gods will punish me [ ]  3. Community elders will punish me [ ]  4. Others (Specify)……………………… | 1  2  3  4 |
| Q32 | Why do you prefer delivering outside health facility? | 1. Because of privacy [ ]  2. Due to traditional beliefs/cultural practices [ ]  3. Others (Specify)………………………………… | \| 1 \| \| --- \| \| 2 \| \| 3 \| |
| Q33 | Are there any religious beliefs that can affect your choice of place of delivery? | 1. Yes [ ]  2. No [ ] | 1  2 |
| Q34 | What are the religious beliefs that may affect your choice of delivery assistance? |  |  |
| Q35 | Do your mother-in-law determine the place of your delivery? | 1. Yes [ ]  2. No [ ] | 1  2 |
| Q36 | What would be the consequences if you seek skilled delivery against the choice of your mother-in-law? |  |  |
| Q37 | Are there any taboos that prohibit you from giving birth at health facility? | 1. Yes [ ]  2. No [ ] | 1  2 |
| Q38 | What are the taboos that prohibit you from giving birth at health facility? |  |  |

**THANK YOU**
